# Psychiatric Symptoms in Patients with Cerebral Endometriosis: A Case Report and Literature Review

**DOI:** 10.3390/jcm11237212

**Published:** 2022-12-04

**Authors:** Camilla Elefante, Giulio Emilio Brancati, Elene Oragvelidze, Lorenzo Lattanzi, Icro Maremmani, Giulio Perugi

**Affiliations:** 1 Psychiatry Unit, Department of Clinical and Experimental Medicine, University of Pisa, 56126 Pisa, Italy; 2 Department of Psychiatry, Tbilisi State Medical University, 0186 Tbilisi, Georgia; 3 Psychiatry Unit, Azienda Ospedaliero-Universitaria Pisana, 56126 Pisa, Italy; 4 G. De Lisio Institute of Behavioral Sciences, 56127 Pisa, Italy; 5 Association for the Application of Neuroscientific Knowledge to Social Aims (AU-CNS), 55045 Pietrasanta, Italy; 6 Saint Camillus International University of Health and Medical Sciences (UniCamillus), 00131 Rome, Italy

**Keywords:** cerebral endometriosis, neuropsychiatry, bipolar disorder, mood instability, brain lesions, frontal lobe, executive functions, neuroimaging

## Abstract

Endometriosis is a systemic medical condition characterized by endometrial tissue that is abnormally implanted in extrauterine sites, including the central nervous system. In this article, we reported the case of a patient with presumed cerebral endometriosis who was diagnosed with bipolar disorder and panic disorder and systematically reviewed the literature for previously reported neuropsychiatric symptoms in patients with cerebral and cerebellar endometriosis. The PubMed, Scopus, and Web of Science bibliographic databases were searched according to the PRISMA guidelines. Seven previous case reports were found and described. While neurological disturbances dominated the clinical picture in the cases retrieved from the literature, our patient represented the first case to show both neurological and psychiatric manifestations. Atypical features of bipolar disorder including chronic mood instability, mixed episodes, and excitatory interepisodic symptoms were highlighted. During the neuropsychological evaluation, a dysexecutive profile consistent with frontal lobe pathology was evidenced. We hypothesized that the course and features of the illness were largely influenced by the presence of documented brain lesions compatible with endometrial implants, especially in the frontal region. Accordingly, patients with endometriosis who exhibit neurological as well as mental symptoms should be investigated for cerebral lesions.

## 1. Introduction

Endometriosis is a gynecological pathology in which the glands and stroma of the endometrial mucosa are abnormally implanted in locations other than uterine cavity and respond to hormonal changes with microscopic internal bleeding, subsequent inflammatory response, neovascularization, and fibrosis [1]. It is most commonly diagnosed in women of reproductive age, usually between the ages of 25 and 29 years old [2], although the disorder can also emerge in the teenage years [3]. It has a prevalence rate of 25–50% in infertile women [4] but can affect as much as 71–87% of women with chronic pelvic pain [5]. Menopause, whether spontaneous or induced, usually leads to the resolution of symptoms [6].

Ectopic endometrial tissue implants are typically located in ovaries, uterine ligaments, recto- and vesicovaginal septum, pelvic peritoneum, cervix, labia, and vagina [7,8]. Uncommon implantation sites include laparotomy scars, pleura, lung, diaphragm, kidney, spleen, gallbladder, nasal mucosa, stomach, breast, eyes, and nervous system [9,10]. Central nervous system involvement is rare compared to peripheral. The majority of cases of central nervous endometriosis affect the conus medullaris and/or cauda equina, but lesions in the brain have also been described [11]. Accordingly, symptoms can vary and include dysmenorrhea, dyspareunia, dyschezia, dysuria, pelvic pain, bloating, nausea, vomiting, hematuria, irritable bladder syndrome, and neurological symptoms [12,13,14].

Endometriosis can be treated medically or surgically. Medical treatment of endometriosis is based on progestins, combined estrogens/progestins, danazol, gonadotropin-releasing hormone (GnRH) antagonists, or GnRH agonists with or without hormone replacement therapy [15]. In addition, aromatase inhibitors have been studied in the treatment of resistant endometriosis [16]. To improve symptoms and fertility, endometriotic tissue can be removed or destroyed surgically. Despite the effective treatments available, recurrence is extremely common [17,18]. Surgical menopause can be induced through bilateral oophorectomy in patients who require it [19].

The high recurrence rate and the involvement of so many different organs and systems favor the notion that endometriosis is a systemic disorder. Recent insights indeed suggest that endometriosis may result in generalized inflammation and metabolic changes that also involve the brain [20]. The term “endometriosis brain” has been coined to describe the combination of structural and functional cerebral changes associated with endometriosis [21,22,23]. Gray-matter volume changes and altered gene expression have been observed, especially in regions involved in pain processing as well as in emotion regulation, cognition, and motivation, which possibly explains the development of psychiatric symptoms [20]. Such brain alterations are unlikely to be solely attributed to endometriosis-related systemic processes. Chronic pelvic pain indeed may represent a risk factor per se for the emergence of psychiatric disorders that is reflected by specific brain changes [24,25]. Hormonal medications such as GnRH-receptor agonists and contraceptives may also contribute to mood instability and depression [26,27]. Alternatively, unknown etiopathogenetic mechanisms may be shared between endometriosis and certain psychiatric conditions.

Depression, anxiety disorders, bipolar disorder, substance use disorders, and attention-deficit/hyperactivity disorder occur more frequently in patients with endometriosis than in controls [28,29,30]. In addition, women with a history of psychiatric problems are more likely to be diagnosed with endometriosis [30]. Bipolar disorder in particular seems to be more specifically related to endometriosis than other conditions. Notably, the prevalence of bipolar disorder (but not that of depression) is higher in women with endometriosis than in those with chronic pelvic pain due to other disorders [23,31]. This association may be hypothetically explained by shared alterations in the susceptibility to hormonal fluctuations [32].

Finally, since mental changes may sometimes be the only symptoms of intracranial lesions [33,34], neuropsychiatric symptoms in patients with endometriosis could also potentially stem from cerebral endometriotic lesions. Nevertheless, no cases of psychiatric disorders associated with cerebral endometriosis have been described so far.

In this report, we describe a patient with presumed cerebral endometriosis who was admitted to our psychiatric ward due to a suicide attempt and diagnoses of bipolar disorder and panic disorder. The patient had a diagnosis of endometriosis and suffered from mood symptoms for more than 25 years. Atypical features and a chronic-fluctuating course complicated the clinical picture and were hypothetically related to ectopic endometriotic tissue lesions in the patient’s brain. The literature on cerebral and cerebellar endometriosis was systematically reviewed to search for previously reported neuropsychiatric symptoms.

## 2. Case Report

Mrs. L.B. is a 50-year-old woman with bipolar disorder, panic disorder, and a history of endometriosis who was first admitted to our hospital at the age of 46 as an inpatient due to a suicide attempt during a hypomanic episode with mixed features. Her first-degree family history was positive for depressive disorders on the maternal side. Cyclothymic temperamental traits and increased emotional reactivity were reported since adolescence.

The patient’s gynecologic history was characterized by the onset at the age of 23 of dysmenorrhea and abdominal–pelvic pain that worsened during menses. The laparoscopy performed a year later showed an endometrioma in the right ovarian and four intestinal endometrial nodules. The ectopic endometrial lesions were surgically removed, and the diagnosis of endometriosis was confirmed via histologic examination. In the following years, the patient had multiple relapses of dyspareunia, pelvic pain, and intestinal diseases, and these symptoms lost their temporal relationship with her menstrual cycle. Different treatment regimens with estro-progestin or progestin pills were prescribed without significant beneficial effects on pain and gynecological symptoms. At the age of 28, the patient started GnRH analogues, but after some months the therapy was interrupted due to the onset of psychiatric symptoms. Overall, the patient had seven interventions for removing endometrial ovarian and intestinal cysts before then undergoing a total hysterectomy with a bilateral oophorectomy at the age of 45.

While the patient’s subclinical mood fluctuations dated back to early adulthood, her syndromal psychiatric symptoms first occurred at 28 years. At that time, in fact, during the treatment with GnRH analogues, she developed depression, panic attacks, avoidant behaviors, anticipatory and separation anxiety, that required temporary psychopharmacological treatment with antidepressants and benzodiazepines. In subsequent years, the patient reported slight fluctuations in mood and energy levels not requiring psychopharmacological treatment and not associated with relevant changes in global functioning. At the age of 32, the patient developed her first hypomanic episode, which was characterized by irritability, hyperarousal, insomnia, agitation, disinhibition, and excessive spending, then followed by a relapse of anxious and depressive symptoms. In order to improve her mood symptoms, psychiatric treatment with gabapentin and sertraline was introduced. Despite an initial improvement, the patient’s affective instability then increased. In the following years, frequent mood swings were described with rapid transitions from hypomanic episodes characterized by mixed features, generalized anxiety, panic attacks, emotional lability, and increased interpersonal sensitivity, to atypical depressions characterized by volitional inhibition, asthenia, emotional incontinence, and disrupted circadian rhythms. Despite multiple treatments with mood stabilizers (e.g., lithium carbonate, valproic acid, lamotrigine, and oxcarbazepine) and antidepressants (e.g., paroxetine, escitalopram, venlafaxine, duloxetine, trazodone, and mianserin), mood episodes recurred often and subsyndromal symptoms of mood lability, cognitive and emotional impulsivity, distractibility, and planning and organizational difficulties persisted between major episodes, causing moderate impairments in working and social functioning for the patient.

Neurological symptoms were also reported from the age of 35. Beginning at that age, the patient experienced paresthesia to the right upper limb and frequent dull and pressure-like bilateral headaches in the orbito-fronto-temporal region that were associated with episodes of impairment of vision, vomiting, and dizziness. Headaches were initially related to menses, but the patient subsequently lost this temporal relationship. Since the headaches were resistant to therapy, she was hospitalized in a neurological unit where clinical and instrumental examinations were performed. Electroencephalography (EEG) and brain magnetic resonance imaging (MRI) were performed. While the EEG was unremarkable, the brain MRI revealed two focal lesions with a malacic center. Both lesions were in the subcortical white matter of the left hemisphere: one in the anterior frontal region and one in the postero-inferior parietal region. The lesions were identified as postischemic, but due to their ambiguous character, the diagnosis was considered “dubious”. In addition, the patient had hemosiderin deposits in the right hemisphere near the uncus and in the frontal subcortical region.

**Figure 1:**
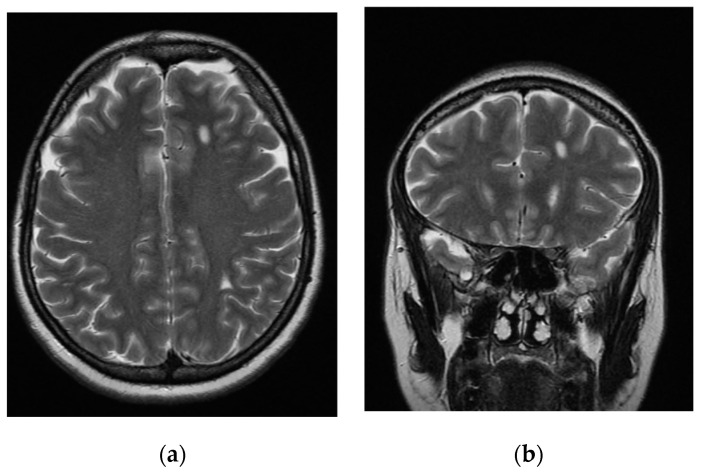
Axial (**a**) and coronal (**b**) fast-spin echo T2-weighted magnetic resonance imaging (MRI) showing two focal gliotic lesions in the subcortical white matter of the left hemisphere in the anterior frontal region (**a**,**b**) and in the postero-inferior parietal region (**a**).

Notably, after iatrogenic menopause, the patient’s gynecological and neurological symptoms remitted while the psychiatric ones did not. At the age of 46, during a hypomanic episode with anxious features, the patient started misusing benzodiazepines out of her medical prescription and had a car accident that resulted in head trauma with a loss of consciousness. She was admitted to the internal medicine ward and underwent computerized tomography (CT), which revealed no acute focal lesions but reported hypodense lesions in the left subcortical inferior parietal region and in the body of the left caudate nucleus. A few weeks after discharge, her mood symptoms worsened. Mrs. L.B. attempted suicide via an intentional drug overdose (pregabalin and delorazepam) and was hospitalized in our psychiatric unit. When she was admitted, a depressive mood, low energy, and crowded thoughts were reported. Bouts of unprovoked self-directed rage and inner tension occurred, and rapid temporary mood elevations characterized by cheerfulness, increased talkativeness, and social disinhibition were also present. While a tendency toward clinophilia was recorded, no motor retardation nor agitation were observed. During the hospitalization, a brain MRI was again performed. The same lesions reported in the previous MRI were confirmed; however, malacic centers had turned into two areas of gliosis (Figure 1). Upon neurological consultation, it was hypothesized that the lesions were cerebral endometriotic foci that had become gliotic following iatrogenic menopause. After examining the patient, the gynecologist suggested that the lesions were consistent with a diagnosis of cerebral endometriosis.

A combination therapy of lithium sulfate (122.5 mg/day), pregabalin (150 mg/day), fluoxetine (10 mg/day), and trazodone (25 mg/day) was introduced with a good response. During follow-up, mild affective lability and impulsivity persisted, so small dosage changes were applied (lithium sulfate up to 166 mg/day and fluoxetine up to 20 mg/day). After two years, the patient had a minor depressive relapse and was again admitted to our inpatient ward. Valproic acid (up to 450 mg/day) was added to the treatment, while pregabalin was discontinued. Upon follow-up, memantine at 10 mg/day was also introduced with additional benefits regarding mood stability, organization, and planning. No more hospitalizations nor major episodes occurred during the subsequent two years. However, the patient’s executive deficits persisted and were supported by neuropsychological testing.

Mrs. L.B. was recently administered 10 subtests of the Wechsler Adult Intelligence Scale—Fourth Edition (WAIS-IV) [35]. Her general cognitive ability as estimated by the Full-Scale Intelligence Quotient (FSIQ) was on the upper bound of the borderline range (FSIQ = 79). However, the FSIQ was unlikely to represent a cohesive, unitary set of abilities based on the patient’s highest (Max) and lowest (Min) scores for the four WAIS-IV indexes compared to the Italian population (Max − Min = 39) [36]. Indeed, while verbal comprehension and perceptual reasoning abilities were both in the average range (Verbal Comprehension Index = 100 and Perceptual Reasoning Index = 92), the patient’s ability to sustain attention, concentrate, and exert mental control was in the borderline range (Working Memory Index = 72), and her ability to process simple or routine visual material without making errors was in the extremely low range (Processing Speed Index = 61). Accordingly, while the General Ability Index (GAI), which is a measure of overall intellectual ability alternative to the FSIQ and is based on the general assessment of fluid and crystallized intelligence, was in the average range (GAI = 96), the Cognitive Proficiency Index (CPI), which is a measure of the effectiveness of cognitive information processing and management, was in the extremely low range (CPI = 66) [37].

## 3. Literature Review

### 3.1. Materials and Methods

#### 3.1.1. Search

A systematic review of the literature was conducted, and the Preferred Reporting Items for Systematic Reviews and Meta-Analyses (PRISMA) guidelines [38] were used to describe the procedures and results. The PubMed, Scopus, and Web of Science bibliographic databases were searched from their date of inception to 22 March 2022. The reference lists of the included studies were also carefully searched for relevant citations. The research team discussed and reviewed the results of an initial scoping search. We developed a strategy using two groups of search terms: “endometriosis” (group 1) and “cerebral” or “cerebellar” or “nervous” or “brain” (group 2). Terms were adapted as necessary for each database. The results were downloaded into Mendeley software (version 1.19.8, Elsevier, Amsterdam, The Netherlands).

#### 3.1.2. Eligibility Criteria

The search included reviews and original studies. Only original studies, case reports, or case series were eligible for our review. If a previous review was found, we searched the reference list to identify and retrieve the primary studies. No restrictions on the study design or group comparisons were applied. In order to be included in our review, study participants or subgroups of participants should have been diagnosed with documented or presumed cerebral or cerebellar endometriosis based on clinical and histological and/or neuroradiological findings. No restrictions on age on symptoms were applied.

#### 3.1.3. Abstract Screening and Study Selection

Using our search strategy, 919 abstracts were retrieved, of which 246 were removed as duplicates and 673 were screened. If a title appeared to be potentially eligible but no abstract was available, the full-text article was retrieved. Two researchers (C.E. and E.O.) scanned all titles and abstracts to identify the relevant articles for full-text retrieval. Disagreements were resolved through discussion after consulting a third reviewer (G.E.B.). A total of 664 records were excluded based solely on the title or abstract. A total of nine full-text articles were thoroughly assessed for eligibility. No additional records were identified through other sources (citations in reference lists of screened papers and reviews).

### 3.2. Results

Seven case reports were included in the systematic review; one was excluded because it described a patient with a recurrent cryptogenic subarachnoid hemorrhage that possibly was due to ectopic endometrial tissue in the spinal canal [39] and the other because it focused on endometriosis patients’ neurological abnormalities caused by chronic pain, mental comorbidities, or endometriosis itself. Seven other documented or presumed cases of patients with cerebral endometriosis were collected and summarized from the literature (Table 1).

No cases included psychiatric disorders, and neuropsychiatric symptoms were reported only in the case of 39-year-old woman with a 3-year history of hallucinations in the context of catamenial epilepsy [44]. In that case, the anamnesis reported a diagnosis of pelvic endometriosis. The epileptic symptoms were temporally connected to the menstrual cycle. The T2-FLAIR MRI scans of the brain showed the presence of hemosiderosis deposits in the globus pallidus. The patient was treated with progestin without any improvement. GnRH analogues were then introduced with complete remission of gynecological, neurological, and psychiatric symptoms. Nevertheless, the treatment was suspended due to many side effects. After discontinuation, the symptoms relapsed, so a selective progesterone receptor agonist (dienogest, 2 mg daily) was then prescribed. The patient’s chronic pelvic pain, dysmenorrhea, and catamenial epilepsy were all completely and permanently resolved 24 weeks after the commencement of the treatment.

Generalized or focal seizures were commonly reported. In three other cases, they were temporally correlated with the menstrual cycle. Ichida et al. reported the case of a 31-year-old woman who had partial seizures on the first day of her menstrual cycle [41]. For around half of the first day of the cycle, the seizures occurred every 30 min. At the time of the seizures, she had a feeling of vague numbness and coldness in the left lower extremity. Multiple interictal electroencephalogram (EEG) recordings revealed well-developed and well-organized alpha background activity without epileptic discharges. Three ring-enhanced lesions were found on a CT scan in the precentral and postcentral gyrus. The T2-weighted images of the brain MRI showed central areas of decreased attenuation in the cysts’ core regions that were indicative of a previous hemorrhage. The patient underwent a biopsy through a right frontoparietal craniotomy. A histological evaluation of the lesions confirmed the presence of endometrial glands and stroma. After surgery, she received 600 mg danazol per day and had no more seizures.

Vilos et al. described a 41-year-old woman who was initially seen for chronic pelvic pain, menorrhagia, and dysmenorrhea [43]. For these symptoms, the patient underwent a combined laparoscopy and hysteroscopic endometrial ablation. Two months after the surgery, she began to manifest paroxysmal headaches with lancinating sensations on the right side of her face at a rate of 20 episodes or more each day. These symptoms were accompanied by focal seizures. The patient experienced sensory motor abnormalities in her arm and sporadic twitching of her right forefinger. Importantly, the symptoms were associated with her menstrual cycle. A brain CT and MRI showed a circumscribed abnormality in the left centrum semiovale of the brain. An EEG was performed that showed no abnormality. However, carbamazepine was prescribed for the focal seizures. The patient underwent an endometrial balloon ablation to decrease her menorrhagia. Despite a decrease in menstrual bleeding, neurological symptoms continued to occur in relation to her menses. A GnRH analogue was administered monthly for 3 months, during which her neurological symptoms disappeared. When the medication was discontinued and her menstruation resumed, her headaches and focal seizures appeared again. Due to the patient’s reluctance to undergo surgical intervention, she was treated with antiepileptic drugs for the following two years. Finally, she agreed to the procedure, and a laparoscopic hysterectomy and salpingo-oophorectomy were carried out. Following the surgery, the cyclical neurological problems disappeared.

One additional case of a 44-year-old woman with epilepsy related to cerebral endometriosis was recently reported [46]. Since menarche, the patient had experienced catamenial epileptic seizures, dysmenorrhea, pelvic discomfort, and dyschezia. She underwent laparoscopic surgery for the excision of a unilateral ovarian endometrioma at the age of 18. She had a pregnancy after which the frequency of seizures increased. The epileptic symptoms did not respond to antiepileptics, estroprogestin, or progestogen-only pill treatments. Due to the treatment resistance, the patient required repeated hospitalizations in an intensive care unit. However, her brain CT and MRI scans did not show any endometriotic lesions. At some point, she developed ocular disorders and sensory motor symptoms that affected her lower right limb. Despite these symptoms, an EEG did not show any anomalies. Treatment with GnRH analogues then resulted in a significant reduction in the number and duration of the patient’s seizures; 18 months later, a salpingo-oophorectomy was performed with a further decrease in the seizure frequency. The patient may have had an undiscovered endometrial implant site in her brain. Alternatively, her catamenial epilepsy could have been caused by hormonal fluctuations.

In other cases, seizures were not related to the phase of the menstrual cycle. The first case, which was described in 1987 [40], was a 20-year-old woman without a medical history of gynecological pathologies who was hospitalized due to generalized seizures and severe occipital headaches. She had a 3-year history of pulsatile occipital headaches that lasted 4–5 min and occurred once or twice each month without any correlation with the menstrual cycle. A CT scan of the brain showed a hypodense ring-enhanced circumscribed lesion localized peripherally in the posteroinferior parietal right lobe. The patient underwent a right parietooccipital craniotomy in which a cystic lesion was observed. Aspiration of the cyst produced a chocolate-brown fluid. Endometrial epithelium and hemorrhagic endometrial stroma were found in the cyst upon histological examination. After the surgery, the patient was treated with danazol at 800 mg/day for six months. She stopped experiencing headaches and did not have any neurological issues at follow-up.

Moreover, Meggyesy et al. described the case of a 39-year-old woman that had undergone multiple operations due to infratentorial brain cysts with consecutive chronic hydrocephalus [45]. She had never experienced neurologic symptoms during her menses. At the age of about 20 years, she had premature amenorrhea. A cyst in the fourth ventricle and syringomyelia to L1 was discovered when she was 27 years old. She progressively developed gait and speech deficits. At the age of 35, she experienced a status epilepticus lasting 40 min. A brain MRI revealed several cysts in the infratentorial region that compressed the brainstem. The patient had increasing speech and swallowing difficulties. A posterior fossa decompression was performed, and the cysts were opened and partially resected. The histology revealed cerebellar endometriosis. Despite the various surgeries, the patient passed away at the age of 39 from complications related to cyst recurrence and hydrocephalus.

Finally, one case with no seizures was described [42]. This was the case of a woman with a medical history of congenital hydrocephalus and multiple shunt revisions since childhood. At the age of 40, she developed a gait disturbance and headaches. A shunt blockage was suspected, but a brain CT scan revealed no increase in the size of the ventricles. Neuroimaging showed a lobulated superior vermian cystic mass with fluid–fluid levels with a component projecting into the superior cerebellar cistern. She underwent surgery to remove the cyst. The cyst contained chocolate-colored fluid. The diagnosis of endometriosis was confirmed by the histological and immunohistochemical analyses, which showed endometrial-type epithelium and hemosiderin-laden macrophages. The neurological symptoms of the patient markedly improved after the surgery.

## 4. Discussion

All the cases collected, including our case, concerned women who showed an onset of neurological or psychiatric symptoms during the childbearing age (20–44 years). All cases found in the literature review were described by specialists in the neurological or gynecological field, including four clinical cases described by neurologists [40,41,42,45], two provided by gynecologists [44,46], and one by a gynecologist together with a neurologist [43]. While neurological symptoms dominated the clinical picture in most cases, psychiatric disorders were never reported. In only one case, hallucinations were described as a cognitive seizure manifestation [44]. 

Cerebral endometriosis has been identified with certainty in four woman via the histological analysis of the lesions after surgical removal [40,41,42,45]. In two of these cases, brain MRI findings that were suggestive of cerebral endometriosis had been previously discovered. The “shading sign” and the existence of fluid–fluid levels were found in these women [41,42]. Despite a lack of studies that defined neuroimaging features of cerebral endometriosis and the inability to make a conclusive diagnosis based on the neuroimaging findings, neuroimaging results such as those mentioned above may strongly suggest endometriosis.

In three cases as well as in ours, the diagnosis of endometriosis was solely presumptive [43,44,46]. In some cases, the presumptive diagnosis was supported by the temporal correlation of neurological symptoms with menstruation and by the fact that the symptomatology was resolved with medical or surgical menopause. Furthermore, in two patients with presumed endometriosis, neuroimaging revealed abnormalities that were compatible with endometriosis [43,44]. In one case, there were gadolinium non-enhancing lesions on the brain MRI [43], and in the other, there were hemosiderin deposits [44]. Instead, in the last patient with presumed endometriosis, an MRI revealed abnormalities that were considered compatible with hypoxic-ischemic episodes [46].

Among patients for which EEG findings were reported, only one had abnormalities at the time of registration. Specifically, this patient had a moderate generalized slowing that was most pronounced over the area of the lesion [40]. For all others, including the patient we described, the EEG was normal and did not appear diriment.

All patients, including the one we described, had neurological symptoms at some point during the course of the disease. Abortions or full-term pregnancies before experiencing symptoms occurred in five out of seven women that were described in literature [40,41,43,46] but not in our patient. The most commonly reported neurological symptoms were seizures and headaches. In fact, six patients found in the literature review had focal or generalized seizures [40,41,43,44,45,46] and three had headaches [40,42,43]. Two patients had sensory motor problems [43,46], and one patient had an ocular disorder [46]. Both of the two individuals with cerebellar endometriosis, as could be predicted, exhibited gait disturbances [42,45]. In four cases, the neurological symptoms were related to menstruation [41,43,44,46].

The treatment of cerebral endometriosis in older cases consisted of the surgical removal of brain lesions with or without further medical treatment with danazol, a synthetic steroid and pituitary gonadotropin inhibitor [40,41,42]. Among the most recent patients, instead only one with a history of chronic hydrocephalus had been treated with brain surgery. This patient did not respond well to the treatment, probably due to a late diagnosis of endometriosis. Unfortunately, she experienced frequent recurrences of cerebral endometriotic cysts up to her death [45]. GnRH analogues were initially employed to treat two individuals. In one case, this medication did not completely resolve the neurological symptoms [46], while in the other it was poorly tolerated [43]; both patients underwent an oophorectomy with [46] or without antiepileptic therapy [43]. Another patient was treated with dienogest, a synthetic steroid [44].

The patient we described had neurological symptoms (but not seizures); however, these were only initially related to menstruation. She had headaches as well as impairment of vision and sensory–motor symptoms. Her psychiatric symptomatology, which was consistent with the diagnosis of bipolar disorder and panic disorder, also seemed to be partially determined by the presence of brain lesions. Particularly, persistent mixed/excitatory symptoms such as lability, irritability, and impulsivity, as well as a dysexecutive neuropsychological profile, which were suggestive of prefrontal cortex impairment, were observed. The MRI revealed a malacic center of the lesions that, consistent with the diagnosis of cerebral endometriosis, became gliotic after gynecological menopause. Hemosiderin deposits were also suggestive of endometriosis. While the neurological symptomatology appeared to have been temporally limited to the period when the brain lesions had just arisen and still had a malacic center, the patient’s psychiatric symptoms persisted over time. The affective illness had a chronic-fluctuating course from the onset, which occurred a few years before the brain lesions became neurologically symptomatic. In this case, estro-progestin and progestin therapies were ineffective, and GnRH analogues were poorly tolerated. Interestingly, an oophorectomy resolved her gynecological and neurological symptoms, while her psychiatric symptoms only attenuated some years after. Notably, the patient had a cyclothymic temperament and a positive family history of mood disorders, which suggested that factors other than cerebral endometriosis may have been related to the occurrence of bipolar disorder. However, we hypothesized that while her psychiatric symptoms were not directly caused by endometriotic lesions, her brain lesions, especially in the frontal regions, may have shaped the features and the course of our patient’s condition. As a matter of fact, it is likely that the brain lesions—both in their early phases and once they became gliotic—were able to considerably worsen the instability of the pre-existing mood illness, e.g., by altering fronto-striatal connections involved in the pathogenesis of affective disorders. In particular, the frontal lesion might have been responsible for the deficits in executive functioning and emotional regulation that persisted between major mood episodes. The chronic nature of the symptoms without intervals of normality, in fact, is atypical for bipolar disorder and has been found in subjects with cerebral masses such as brain tumors [47]. Late-onset mania or hypomania, mood disorders with atypical course, unremitting mania, and a poor response of manic symptoms to antimanic treatment were related to the presence of focal brain lesions in previous studies [34,47]. Mania and manic-like symptoms may indeed arise from focal brain lesions that affect the frontal, temporal, and subcortical limbic brain regions and their connecting circuits [47,48]. Although lesional mania has a preferential association with right-hemisphere lesions [34,49], chronic mania and rapid cyclic bipolar disorder have been described in patients with focal lesion in the left hemisphere [33,50,51,52].

## 5. Conclusions

Endometriosis has been linked to a high rate of mood disorders, especially bipolar disorder. Women with endometriosis have a higher risk of developing psychiatric disorders due to problems secondary to endometriosis such as chronic pain, side effects of treatments, altered susceptibility to hormonal fluctuations, altered gene expression, or gray-matter lesions in key regions for mood regulation. In addition to the causes mentioned above, we suggest that cerebral endometriosis may contribute to the onset and course of psychiatric disorders in patients with endometriosis. While some cases of neurological disturbances that emerged due to cerebral endometriosis were previously reported, we described the case of a patient with two brain lesions that presumably were due to endometriosis and whose bipolar disorder was characterized by a pattern of frequent mood swings with mixed features and excitatory/dysexecutive interepisodic symptoms. Despite it being generally acknowledged that brain injuries can cause neuropsychiatric symptoms, these may go frequently unrecognized in individuals with endometriosis. Compared to neurological manifestations, the relationship of psychiatric symptoms with menstruation and menopause may in fact be less obvious. Especially when clinical reasoning leads to uncertain interpretations, brain imaging may become essential for a proper diagnosis. Although there are no MRI-specific features of cerebral endometriosis, some findings may be very suggestive of the disease and can support the diagnosis. Accordingly, cerebral endometriosis should be investigated in patients with endometriosis who show neurological as well as psychiatric symptoms.

## Figures and Tables

**Table 1:** Case reports of patients with documented or presumed cerebral or cerebellar endometriosis.

	Age	Gynecological History	Neurological Signs or Symptoms	Instrumental Examinations	Lesion Site(s)	Findings of the Lesion(s)	Histological Diagnosis	Catamenial Symptoms	Treatment	Remission
Thibodeau et al., 1987 [40]	20	Normal menstrual history; 2 therapeutic abortions	Generalized seizure; 3-year history of 4–5 min occipital headaches	CT, EEG, cerebral angiography	One lesion in the posteroinferior parietal right lobe	Hypodense ring enhancing well circumscribed lesion on CT; moderate generalized slowing, especially over the right temporal area on EEG	Yes	No	Brain surgery and danazol 800 mg/day for 6 months	Yes
Ichida et al., 1993 [41]	31	Regular menses; 2 pregnancies	Partial seizures once every 30 min for around half of the first day of the cycle	CT, MRI, EEG, cerebral angiography	2 lesions in precentral gyrus, 1 lesion in postcentral gyrus	3 ring-enhancing lesions on CT; T2-weighted MRI images revealed central areas of decreased attenuation	Yes	Yes	Brain surgery and danazol 600 mg/day for 6 months	Yes
Sarma et al., 2004 [42]	40	No history of pelvic pain or infertility; 1 caesarean birth	Gait disturbance, headaches	CT, MRI	1 multiloculated cystic mass arising from cerebellar vermis	Mild rim enhancement, shading sign, and fluid–fluid levels on MRI	Yes	No	Brain surgery	Yes
Vilos et al., 2011 [43]	41	Chronic pelvic pain, menorrhagia, dysmenorrhea, and myoma; 3 caesarean births; tubal ligation	Sensory motory abnormalities, pains on the face, paroxysmal headaches, focal seizures	CT, MRI, EEG	Circumscribed abnormality in the centrum semiovale	1 brain lesion without significant gadolinium enhancement on MRI; no abnormalities on EEG	No	Yes	Endometrial balloon ablation, GnRH-a, antiepileptics, salpingo-oophorectomy	Yes
Maniglio et al., 2018 [44]	39	Pelvic endometriosis with dysmenorrhea, dyschezia, and chronic pain	Catamenial epilepsy with hallucinations	MRI	Cerebral hemosiderosis deposits in globus pallidus	Hemosiderin deposits	No	Yes	Progestin therapy, GnRH-a, dienogest	Yes
Meggyesy et al., 2020 [45]	39	Menorrhagia and amenorrhea	Chronic hydrocephalus, gait disturbances, epilepsy, progressive neurological impairment causing death	MRI	1 single cyst in the fourth ventricle, then multiple infratentorial cysts	No fluid suggestive of intracystal bleeds on MRI	Yes	No	Brain surgery, hormonal treatment	No (cyst recurrence)
Antonio et al., 2021 [46]	44	Ovarian endometrioma, dysmenorrhea, pelvic pain, and dyschezia; 1 caesarean birth	Catamential epilepsy, ocular disorder, limb paraesthesia/hypoaesthesia	CT, MRI, EEG, liquor examination	Not found	Not found	No	Yes	Hormonal treatment, GnRH-a salpingo-oophorectomy, antiepileptics	Yes

CT: computerized tomography; EEG: electroencephalogram; MRI: magnetic resonance imaging; GnRH-a: gonadotropin-releasing hormone analogues.

## Data Availability

Not applicable.
